# A conceptual framework for training of trainers (ToT) interventions in global health

**DOI:** 10.1186/s12992-018-0420-3

**Published:** 2018-10-22

**Authors:** Maru Mormina, Sophie Pinder

**Affiliations:** 1 Faculty of Humanities and Social Sciences, University of Winchester, Winchester, UK; 2 Tropical Health and Education Trust (THET), London, UK

**Keywords:** Capacity building, Global Health, Health systems strengthening, North-south partnerships, Training of trainers

## Abstract

**Background:**

Global health partnerships (GHP) between high or low-middle income countries are considered one of the best approaches to health systems strengthening. They typically involve highly skilled healthcare workers who volunteer to deliver capacity strengthening projects overseas, often in the form of peer-to-peer support through training and mentoring. Given GHP’s strong focus on education and training, a common assumption is that training of trainers (ToT) is a strong predictor of sustainability because of its potential for up-skilling the workforce rapidly, cheaply and exponentially by developing local educators. Our aim is to test this assumption and identify the strengths and limitations of this approach by analysing qualitative data from a set of GHP funded by the UK Department for International Development through the Tropical Health and Education Trust.

**Results:**

Our analysis identifies some of the common features of the ToT model and a number of limitations that can prevent it from being both effective and sustainable. Whilst most GHP strive for the long-term sustainability of the training by focusing on adequate training provision and support of local trainers, the wider issues that can facilitate or prevent the continuation of training are not always considered. We propose a conceptual framework (TRAIN) for ToT interventions to help inform practice and project evaluation. We illustrate the applicability of our framework through five case studies, each chosen to illustrate one aspect of the framework.

**Conclusions:**

TRAIN is intended as a starting point for further refinements and discussions about the factors affecting capacity building strategies based on training cascades. Although we envisage its usefulness to GHP as a guidance to design and operationalise ToT, we recognise that in practice it may be difficult to implement it in its entirety. The key message underlying TRAIN is that the sustainability of a cascade depends on a number of factors being present or developing at different operational levels during the course of a project. These are crucial to transform the opportunities that ToT affords to health systems in developing countries into the actual achievement of a training cascade that ultimately upskills the workforce and improves health outcomes in these countries.

**Electronic supplementary material:**

The online version of this article (10.1186/s12992-018-0420-3) contains supplementary material, which is available to authorized users.

## Background

Shortages and poor retention of health workers represent a significant problem of health systems in many developing countries, with major impact on health outcomes [1]. Heavy workloads, poor salaries and limited access to training, education, mentoring and continuous professional development (CPD) [2] are all contributing factors that undermine the morale and commitment of healthcare workers. This, in turn, contributes to complex migration patterns from low and middle income countries (LMICs) to high income countries (HICs) that exacerbate current workforce imbalances [3]. The World Health Organization forecasts that the healthcare workforce deficit in LMICs will reach 12.9 million skilled health professionals by 2035 [4]. For this reason, a strong focus on strengthening the capacity of the healthcare workforce is crucial.

Since the publication of the Crisp report [5], which openly endorsed global health partnerships (GHP) as one of the best approaches to healthcare capacity strengthening, there has been an increased interest and support for this kind of approach to development. GHP are usually defined as long-term, sustainable and usually voluntary collaborations between institutions with similar objectives for the mutual exchange of skills, knowledge and experience [6]. GHP tend to be established between partners in LMICs and HICs, though collaborations between institutions with similar objectives both based in LMICs (South-South collaborations) are becoming increasingly common. They typically involve highly skilled healthcare workers from HICs (or other LMICs) who volunteer to deliver capacity strengthening projects overseas, most commonly in the form of peer-to-peer support through training and mentoring [7]. As with most development initiatives, GHP place strong emphasis in sustainability, which we define here as “the ability of a project to continue to function effectively, for the foreseeable future (…)” [8]. Yet, despite its centrality in project planning and evaluation, there is little evidence to guide GHP on how to achieve it.

Given GHP’s strong focus on education and training, a common assumption is that training of trainers (ToT) is a strong predictor of sustainability. ToT is very popular within the development industry because of its potential for up-skilling the workforce rapidly, cheaply and exponentially. The basic idea of ToT is to initiate a training cascade: skills and knowledge are taught to a small group of trainees who become trainers and go on to transfer those skills to others [9]. Thus, over time, up-skilling through training should become self-sustaining. This, however, assumes that the cascade is allowed to proceed swiftly and unimpeded by other factors, such as attrition among the new trainers or availability of resources for scaling up the training. To presuppose that the problem of staff shortages and limited capacity of the health workforce in LMICs can be solved sustainably by training local trainers seems at best simplistic and warrants closer scrutiny.

Extended funding is rarely given for the evaluation of a partnership [10] and for this reason training is often assessed within a short-term horizon. This constrains the capacity to test the above assumption by asking questions about the effectiveness of the training and crucially about its long-term sustainability (*ibid*). Consequently, there is a dearth of empirical evidence on the topic. Within this limitation, the aim of this paper is first to identify some of the common features of the ToT model, as implemented within a set of GHP funded by the UK Department for International Development (DFID) through the Tropical Health and Education Trust (THET), an international non-governmental organisation that promotes and supports GHP. Informed by our findings and applying Capabilities theory [11], we propose a conceptual framework for ToT interventions to guide practice and project evaluation. We show the applicability of our framework through five case studies, each chosen to illustrate one aspect of the framework. Given the widespread use of ToT in global health initiatives it is important to establish under which conditions this approach can offer a sustainable solution to the capacity problem in the healthcare workforce of LMICs’.

## Methods

In order to characterise ToT initiatives, we analysed GHP in receipt of medium-size grants from THET (typically between £15-30 K). We excluded on-going partnerships and focused only on completed projects to capture some of the longer-term outcomes of a ToT, particularly in relation to the cascading of training. This resulted in a dataset of 15 GHP spread mostly across the African continent with ToT activities covering a wide range of clinical and technical skills (see Additional file 1). The research consisted of documentary analysis (application forms, progress reports, email communications, meeting notes, media reports, academic outputs, etc.), using a directed content analysis. Unlike conventional content analysis, where coding categories are derived directly from the text, the directed approach starts with a theory or relevant empirical evidence as a guidance for deriving initial codes. [12]. In our case, we derived such theoretical and empirical evidence from the academic and grey literature (the latter referring to publications outside traditional academic channels, such as government reports), which we mined using the following keywords: “Training the trainer”, “training of trainers”, “cascade training”, “global health partnerships”, “international development”, either alone or in combination. It is difficult to assess the extent of existing research and other materials relating to ToT, therefore it is possible that some relevant information was not captured in the literature review.

Whenever gaps in the documentary material were found, the analysis was supplemented with key informant interviews; these were typically project leads. An iterative process consisting of several readings of the documents and the wider literature allowed us to derive a set of 32 key variables that we used as codes. These were further refined and grouped into categories and subsequently into themes.

Using Sen’s Capability Approach (CA) as our analytical lens [11], we then conceptualised an evaluative framework that integrates the evidence derived from the content analysis, together with organisational knowledge (i.e. THET’s practical experience), key informant interviews and the analysis of the literature. The CA is a philosophical theory, originally developed by Amartya Sen to counter what he saw as a reductionist evaluation of development purely in terms of access to resources (wealth, income, legal rights, etc.). The CA, instead, focuses on what individuals can be and do (their capabilities), rather the resources available to them. Capabilities are therefore, the opportunities that individuals have to engage in the actions and activities that are important to them. Because capabilities are multi-dimensional, they must be evaluated across multiple domains. For this reason, the CA represents a suitable conceptual tool to articulate a framework that helps us evaluate the extent to which ToT interventions help expand healthcare workers’ capacity beyond a unidimensional focus on skills training.

From our dataset, we selected five case studies for further analysis; for simplicity, each case is here described to illuminate one aspect of the framework. Such single focus inevitably invited omissions and simplifications, and therefore it is possible that our account does not fully capture the complexity and richness of each experience. This is because whilst each case study was chosen to provide a practical example of a specific element of our framework, they all interacted with elements from every part of the framework.

## Results and discussion

### ToT interventions within THET-funded GHP

Across all 15 GHP, a number of common themes emerged. In general, most partnerships have a long history of collaboration and therefore assessment of training needs was almost invariably conducted jointly. Though the ToT initiatives we reviewed were very different and covered a wide range of topics, in general the focus was strongly on skills development of clinical and technical staff. This is unsurprising, given THET’s long history of fostering partnerships among these cadres. Yet, good healthcare management and leadership are also essential for strong health systems [13, 14]; thus, a more active promotion of partnerships to help build leadership capacity was identified as an area requiring further development. At the other end of the spectrum, we observed that frontline community workers were also unrepresented in ToT interventions (and training in general).

In the majority of cases (9/15), ToT was embedded into a wider training plan, thus representing a relatively small element of the project. Potential local trainers (LTs) were generally identified by the LMIC partner; yet, the specific selection criteria were not consistently reported, except for the requirement of relevant clinical expertise. This suggests that the selection of LTs was based largely on an assessment of “hard” technical skills. Assessment of “soft” skills (e.g. enthusiasm, willingness to teach, communication skills) was not routinely reported, suggesting that such skills were either implicitly evaluated or not considered part of the selection criteria. The selection of HIC master trainers (MTs) was also largely based on the assessment of hard skills but, in addition, availability, developing country experience and teaching experience were also consistently considered. The teaching of technical skills was the main focus of most ToT curricula, though pedagogical skills were included in 10/15 ToT projects.

ToT was usually delivered in the early to middle phases of the projects, with gradual transfer of training from the HIC to the LMIC partner towards the latter stages. However, this was not observed consistently across partnerships. The ability of LTs to take ownership of the training seemed influenced by a number of factors, crucially time and resources. ToT is generally delivered in the context of time-bound projects lasting between one and three years, a timescale that may not allow for a full transfer of training skills, particularly when those skills are highly complex and technical. In our dataset, only three ToT projects achieved a complete transfer of training from MTs to LTs; in most cases, however, LTs were still reliant on MT support by the end of the project, thus calling into question the sustainability of the training. LT attrition was generally not captured by end of project reports, in part because these are usually submitted within six months of completion. To appreciate the extent of LT retention, as well as the impact and continuity of the training cascade, such short-term evaluation is unrealistic. Instead, a two-year post-project evaluation may provide a more accurate measurement of this indicator.

Although most of the GHPs analysed were endorsed by national, regional or institutional authorities, in many cases endorsement did not translate into concrete support in the form of financial resources, assistance with recruitment of trainees, etc. Moreover, more than half of GHP in our dataset had no clear plans to roll out the training or to offer long-term support to newly qualified LTs (e.g. through CPD) beyond the initial training given. Hence, cascading of training was highly variable, ranging from none to several hundred health workers being locally trained by the end of the project.

Though caution is required in assessing the above findings due to the small sample analysed, they do nonetheless reveal that various factors outside the specific training activities conducted by partnerships can either enable or hinder a ToT cascade: local conditions, lack of time and resources, inadequate facilities, inadequate institutional support (e.g. for the promotion of training workshops), etc. These factors challenge the assumption that simply increasing training capacity translates in a more capable workforce; we must therefore think critically about ToT beyond this single quantitative dimension.

### The ‘TRAIN’ framework

Given the above findings, we suggest that an effective approach to ToT must consider the multiplicity of factors that impact on the capacity of trainers to sustain a training cascade, beyond their own skillset. A training cascade is a process whose outcome is to strengthen the workforce capacity. ToT planning and evaluation, therefore, requires capturing outcome as well as process measures, the ultimate goal and the steps required to achieve it [15]. One of the difficulties with process evaluation is that it requires different indicators depending on which strategies partnerships use to build capacity [16]. However, irrespective of the specific indicators, the questions that need to be answered cannot be only about outcomes, but fundamentally about the factors that affect people’s capacity to achieve those outcomes.

Because ToT is a process of expanding healthcare workers’ capabilities to realise specific achievements, it is amenable to analysis through the lens of Sen’s CA [11]. In brief, the CA stresses the importance of agency and empowerment. Capabilities are the opportunities open to individuals to realise different “functionings” (resources, activities, attitudes) that they recognise as valuable. What matters from an evaluation perspective is not so much the functionings (i.e outcomes or achievements), but the choice and agency to achieve them and crucially, the conversion factors, i.e. the forces that help or hinder individuals’ capacity to convert capabilities into functionings, or in other words, their ability to turn opportunity into achievement (i.e the process). The CA interests us here precisely because it allows us to develop an evaluative framework for ToT that captures the interaction between individual capabilities (the opportunity set that training offers), achievement (the cascading of training and ultimately the up-skilling of the healthcare workforce) and conversion factors (the political, social and economic determinants at play when building capacity through ToT).

**Fig. 1:**
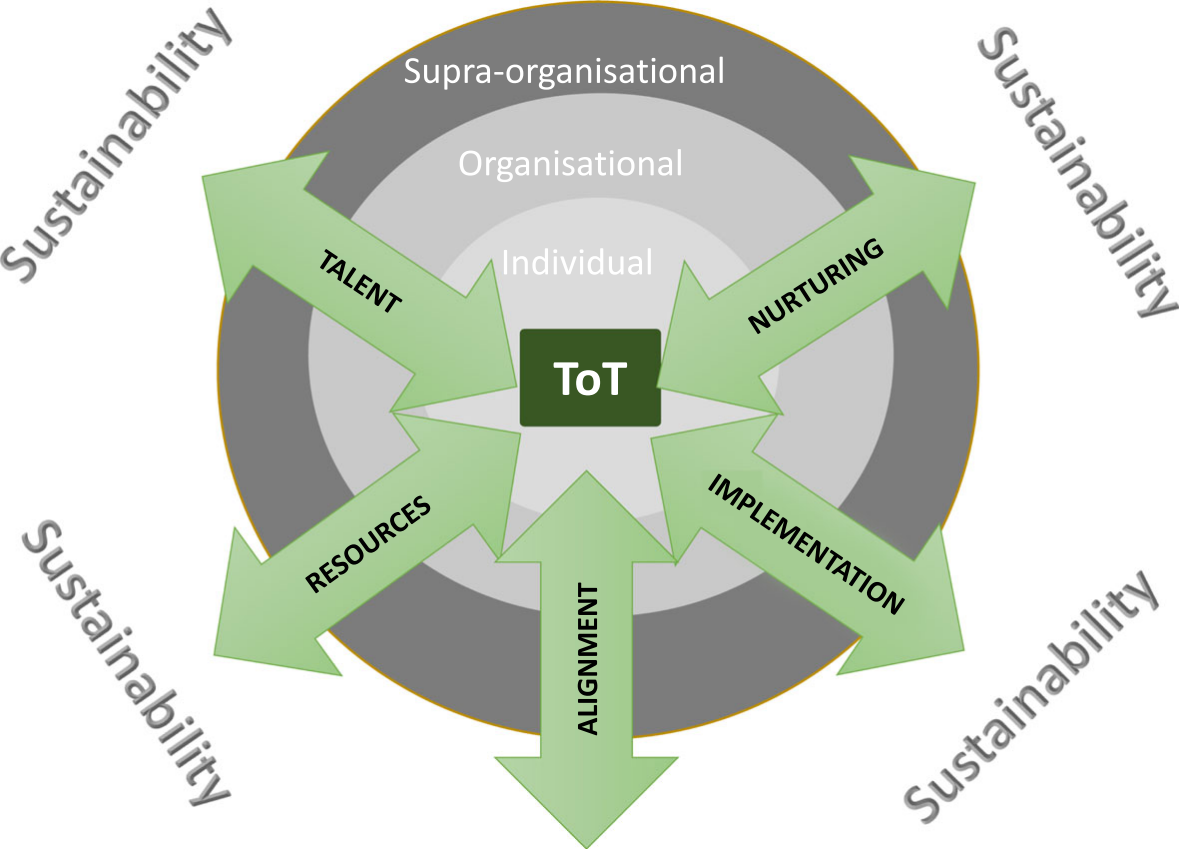
The TRAIN framework

Informed by the philosophy of the CA, we propose an analytical framework, which we call TRAIN. It aims to help practitioners identify the negative and positive influences on ToT in order to develop appropriate and sustainable strategies. TRAIN stands for Talent, Resources, Alignment, Implementation and Nurture, which capture the various elements that need to be integrated if the ToT model is to succeed. The framework (Fig. 1), which is adapted from Cooke [17], has two dimensions:

*Three levels of impact*: these are the individual, organisational and supra-organisational (networks, national and international bodies, etc.)

*Five constitutive elements of ToT*: these are discussed in detail below and are represented in the diagram by arrows that indicate their contribution towards ToT. The arrows traverse the three structural levels suggesting that activities and processes at all levels can influence the contribution of each element to the success of a ToT. The arrows point in both directions to indicate that activities and policies applied at one level can have an impact at all other levels.

Underlying the framework is the notion of sustainability. Whilst this is not directly measured, the framework’s constitutive elements contribute to the self-sustained long-term training capacity of the organisation receiving the ToT intervention.

### Talent

The main goal of a ToT intervention is to train participants to become trainers. Thus, both MTs and LTs must have the right blend of skills, experience and knowledge to train others (talent). A trainer without professional experience (“hard skills”) has nothing to teach, and this is often considered the main attribute of a good trainer. However, equally important is to have a clear conceptual understanding of his/her own practice and the ability to transfer this knowledge to others (“soft skills”). Though individuals can be taught teaching techniques, not everyone is able to teach. Key personal attributes are essential to become a trainer: patience, insight, confidence, communication skills, leadership, capacity for self-reflection, ability to be constructively critical, and above all motivation to help others [9]. In addition, MTs working in LMICs must have cultural awareness and flexibility to adapt training to the local circumstances. LTs’ long-term commitment to train is also critical to avoid the high levels of attrition common in LMICs. Thus, at the organisational level, partner organisations should have procedures in place to identify potential trainers (MTs and LTs), e.g. through pre-recruitment aptitude tests that assess both soft and hard skills. At the supra-organisational level, policies and incentives can be introduced to help talent recruitment and retention, such as professional accreditation, benefit packages, progression paths, etc. Tables 1 and 2 show some examples of criteria that can aid talent assessment for master and local trainers.

**Table 1:** Talent. Master Trainers

STRUCTURAL LEVEL	SUGGESTED CRITERIA (examples)
Individual	Enthusiasm to train Communication/Leadership skills (incl. Cultural awareness) Competence
Organisational	Fair procedure to identify trainers Incentives for trainers (leave, etc.)
Supra-organisational	Incentives for training Policies

**Table 2:** Talent. Local Trainers

STRUCTURAL LEVEL	SUGGESTED CRITERIA (examples)
Individual	Enthusiasm to train Communication/Leadership skills Competence (technical and training) Commitment
Organisational	Fair procedure to identify trainers Incentives for trainers Strategy for developing potential trainers
Supra-organisational	Incentives for training Recognition of trainers (e.g. accreditation by professional bodies)

#### Case 1: Training health Workers in Kenya: Talent selection and collaborative development

In order to strengthen antenatal, intrapartum and postnatal care in four Kenyan regions, a partnership was established between a Teaching Health Board in Wales responsible for delivering healthcare services to rural communities and its Kenyan counterpart. The partnership, consisting of midwives from Wales as well as public health and maternal and sexual health service leads in Kenya, co-delivered a ToT project for local health workers to transfer key knowledge in maternity care to traditional birth referral agents (TBRAs). Special attention was paid to the selection and management of both the Welsh health professionals who would become MTs and the Kenyan health workers identified as potential LTs. This proved crucial to ensure the effective cascading of training at the local level. For the Welsh partner, this meant a rigorous recruitment process in line with the Health Board’s Volunteer Policy to identify and recruit suitable health professionals to design, develop and teach the ToT course to the Kenyan health practitioners. The motivations of prospective MTs for joining the project were specifically taken into account during the recruitment process. Candidates were recruited following extensive discussions with project leads and the Steering Group in which the candidate’s role in the project and the extent of their involvement was clearly defined.

The three Welsh volunteers were all experienced midwives chosen because of their specific skills, as well as their enthusiasm and commitment to the partnership. One of them had significant professional experience as well as project management and evaluation skills gained through a previous role as practice development midwife in the UK. Moreover, she had previously developed training programmes and supported staff to develop skills in maternity care in the Kenyan setting. The two other volunteers were clinical midwives with extensive UK-based experience in midwife-led care and out of hospital births. For their part, the Kenyan management team selected local community health volunteers to attend the ToT course as prospective LTs based on their enthusiasm to work with local communities beyond their usual health care roles and their interest in leading improvements in community health care. Assessing motivation and commitment was essential given that about half of the candidates were unpaid health workers.

Several strategies were embedded in the four-day ToT programme aimed at developing the training potential of prospective LTs. These involved exploring the participants’ experiences and level of knowledge of local community needs, allowing them to reflect on these needs in relation to their new roles and responsibilities as trainers. In addition to clinical skills and knowledge training, trainees were also taught teaching and facilitation techniques, so they could effectively cascade the training to the TBRAs. LTs were encouraged to draw detailed training plans for TBRAs based on the local challenges they identified, thus fostering a sense of ownership. LTs received face-to-face support from MTs to deliver their first training course, with subsequent training activities being supported by remote mentoring.

By the end of the project, all 16 health workers who completed the ToT course had cascaded the training to a total of 106 TBRAs. The locally owned training programme brought into close contact local health workers and TBRAs, and this resulted in increased mutual trust between these cadres: TBRAs made 941 referrals to skilled health workers and the health staff began to value the role of the TBRAs as a local resource to draw upon for support and information at community level. Some of the TBRAs even gained employment at local dispensaries.

### Resources

This rubric includes the structures and processes that enable training to cascade. Time is the main resource limitation reported not just by the partnerships in our dataset but more broadly in the literature [e.g. [18]]. Many LTs struggle to find the time to attend the ToT course and to cascade the training afterwards. MTs also experience time constraints, usually having to fit overseas volunteering during their annual leave or around highly demanding full-time jobs. Releasing staff from their normal duties is crucial, yet often met with reluctance. HIC organisations can perceive this as an unnecessary burden, although the benefits for volunteers in terms of professional development have been richly described [5, 19]. LMIC organisations facing understaffing need to integrate ToT within their wider strategic plans. This can be done by adequately identifying the most suitable LTs and conducting a realistic assessment of the organisation’s capacity to absorb the workload once individuals are released from their duties to become trainers, instead of uncritically welcoming overseas input that might, in the long term, result in undue pressure on the workforce.

Time to develop as a trainer is also important. Most ToT courses are run as short workshops, which may not always be appropriate. If topics are new to trainees or require behaviour changes, longer timeframes may need to be built into the ToT curriculum to allow trainees to learn, assimilate the information and feel ready to teach it to others [9].

Besides time constraints, lack of financial and other material resources can hinder ToT participation and cascade. There is often an unspoken assumption that resource mobilisation by the overseas partner will help cover recurring costs such as refreshments, teaching equipment, travel expenses, etc. The importance of these contributions to the success of a ToT cannot be understated but neither can it be assumed that these will always be covered by the HIC partner. Therefore, shared responsibility with clear commitments and transparent financial arrangements can help reduce barriers to access and avoid imposing undue resource burden on partners. Such commitment should not be limited to financial support. Managers in LMIC institutions are critical enablers of the training cascade, e.g. by helping disseminate information about training events. Poor communication may result in training being cancelled, LTs demotivation and eventually the collapse of the cascade. At the supra-organisational level, facilitators of ToT courses and cascades include financial support, infrastructure (e.g. venues for training events) and dissemination, particularly when the training takes place across institutions or regions. Table 3 shows some examples of criteria for measuring resource availability at different levels.

**Table 3:** Resources

STRUCTURAL LEVEL	SUGGESTED CRITERIA (examples)
Individual	Time to develop as a trainer Time to undertake the training Financial and physical resources (for travel, fees, etc.)
Organisational	Evidence of capacity and willingness to release staff Physical resources allocation(e.g. computers) Local financing of recurrent costs (e.g. per-diems, refreshments) Dissemination and awareness
Supra-organisational	Bursaries or other schemes for financial assistance Dissemination and awareness Accountability and transparency for resource allocation Structures to support training (e.g. training centres)

#### Case 2: Negotiating time and resources for the development of an internationally recognised gastrointenstinal endoscopy training in Malawi

Acute Upper Gastrointestinal Bleed is a common medical emergency requiring timely and appropriate intervention, but its management can be challenging if medical supplies, equipment and trained personnel are inadequate [20]. Following visits and a needs assessment in 2009, a government tertiary teaching hospital in Malawi and a UK university teaching hospital formed a partnership to improve local endoscopy services. The partnership focused on the provision of endoscopy equipment, as well as training and support for endoscopy staff in Malawi. To this end, a trainer from the UK provided hands-on training in basic endoscopy skills to a number of trainee doctors and clinical officers. In addition, the UK team worked with a group of experienced and trainee nursing staff on improvements in practice and protocols.

The training was established to closely mirror the standards of UK gastrointestinal endoscopy courses. This included the requirement that trainees continue training (under a suitably qualified trainer) for a further six months after the completion of the course. This well-founded principle was clearly as important in the Malawian setting as it is in the UK. Yet, lack of properly trained endoscopy trainers in the country became an obstacle for meeting this requirement. Given the interest on gastroscopy training from various hospitals in Malawi, an opportunity was identified, therefore, to develop a Train the Gastroscopy Trainers (TGT) course to provide teaching training and skills-enrichment for consultants. The long-term expectation was to establish a National Endoscopy Training Centre by attracting official recognition and funding both from within the country and from overseas.

The TGT programme was developed to specifically and effectively enhance the teaching skills of the local faculty in a limited timeframe. The training involved local faculty personnel observing UK trainers teaching the TGT, then delivering part of the training themselves with the support and supervision of the UK team, thus simultaneously training new trainers and enhancing the skills of the existing local faculty to deliver future ToT. Of significant concern was the fact that the running of training courses would affect the vital provision of health services by taking staff out of their jobs for periods of time. The partnership addressed this concern by incorporating training practice on live patients and by effective coordination with the hospital to ensure that courses were delivered and staff were released only when service provision could be guaranteed. Such embedding of training activities within the cycle of service provision ensured a positive attitude towards local trainers and good support from their unit and employer.

As a result of the partnership’s ongoing work, in 2015, a Memorandum of Understanding (MoU) was signed between the World Gastroenterology Organisation (WGO), British Society of Gastroenterology (BSG), the course accrediting body in the UK and The Malawi Training Centre to establish an international training endoscopy centre in Malawi’s second largest city, Blantyre. The Centre is jointly managed by LMIC and HIC partners, including Malawi’s College of Medicine, the Malawian Ministry of Health, the Blantyre hospital and a programme funded by the UK’s largest biomedical research charity, the Wellcome Trust. The MoU clearly outlines the division of responsibilities between partners. For instance, the WGO has committed to promote the centre and its courses among its networks, the BSC offers free international membership to local trainer faculty in Malawi and local training centre delegates. In May 2016, the Centre was inaugurated as a WGO training centre, cementing the commitment of international and local partners to jointly develop and run educational programmes through the Centre.

### Alignment

ToT interventions will succeed if they align with individuals’ professional goals. Becoming a trainer, thus, should be recognised as part of a career path and not as an adjunct duty if attrition is to be avoided. GHP are rarely justified if their activities are not tailored to the local context and health needs [21]. Similarly, ToT should feed into local needs and organisational priorities. A good alignment between the outcome of a ToT and the organisation’s strategic objectives is more likely to result in greater buy-in and support for cascade activities from local managers and leaders. Such support can materialise, for example, in successful lobbying at supra-organisational levels to establish policies and processes that facilitate the up-skilling of specific cadres. Alignment with regional or country priorities is also important, and this will require the establishment of formal and informal coalitions of different organisations (hospitals, districts, professional bodies) to influence the policy environment (Table 4).

**Table 4:** Alignment

STRUCTURAL LEVEL	SUGGESTED CRITERIA (examples)
Individual	Training part of individual career path (retention)
Organisational	Training feeds into institutional priorities Management supportive and actively involved Capacity/Willingness to lobby MoU
Supra-organisational	Published health policies Formal and informal coalitions

#### Case 3: Changing practice in maternity care in Zimbabwe

Maternal mortality in Zimbabwe remains among the highest in the world, standing at 443 per 100,000 live births [22]. This motivated the establishment of a partnership between one of the largest hospitals in Zimbabwe and an NHS Foundation Trust in the UK, with the aim to improve the quality of maternity services offered by the former. To this end, a team of midwives, obstetricians, a paediatrician, an anaesthetist from the UK, together with members of a charity dedicated to providing health training in Zimbabwe, travelled to the country in November 2011 to run a Practical Obstetric Multi-Professional Training (PROMPT) ToT course. PROMPT is an evidence based multi-professional training package for obstetric emergencies. The course has been directly associated with improvements in perinatal outcome and enhanced knowledge, clinical skills and team working [23]. The partnership hoped that introducing this training in Zimbabwe would improve outcomes for expectant mothers and save many lives.

From the outset, the partnership engaged the local hospital board and key leaders across the institution in their discussions to set up a maternity quality improvement package of in-hospital training, tools and performance monitoring. Through this early engagement with the institution’s managers, the partnership secured the hospital’s commitment to the project, creating an enabling environment for the training to take place. For example, acknowledging the competing demands on staff time, the Board agreed the release of all maternity staff from their clinical duties to attend a one-day training course at the hospital. Furthermore, it considered the training as part of staff normal duties, thus paying staff their usual salary during the training instead of the usual per diems for off-site training. These are often given as a financial incentive to increase staff motivation to attend meetings or conduct work-related travel but can have a distorting effect on employees’ attitudes [24], for example by prioritising financial gain over professional standards. By removing per diems, the hospital helped cement a new attitude to training, not as an additional duty but as integral part of their professional responsibility for continuous development.

This alignment between hospital policy and the partnership activities enabled the delivery of 10 one-day training courses. In the first year alone, 87% of all hospital staff were trained and by the end of the project, 252 staff members across a range of cadres had been trained, most of whom had never attended any obstetric emergency training. In addition to conducting PROMPT training, Zimbabwean staff made a number of practical improvements to their working environment which enabled a better monitoring of clinical outcomes.

The partnership was also able to engage with an important body running health delivery institutions across the country. Seeing the success of the PROMT training, this body agreed to support the roll out of the training to the 52 health facilities that they own and run across the country. They followed a similar model, making training mandatory for all staff and agreeing to their release from clinical duties without additional per diems. This experience demonstrates the importance of embedding training duties into the career path of health professionals through effective management that aligns hospital policy to training objectives. As the partnership stated, *“a policy removing the requirement for per diems establishes on-going clinical training as the norm within healthcare institutions.”*

### Implementation

ToT evaluation usually involves assessing learning outcomes through post-course questionnaires, i.e. the transfer of skills from MTs to LTs. Yet, the long-term effectiveness of the intervention is often poorly measured. ToT success depends on whether LTs implement a sustainable training cascade, i.e. deliver and develop training courses. A clear implementation strategy (at the organisational or supra-organisational level) is critical to cascade the training. This may mean making training mandatory within an organisation or establishing educational programmes that make use of LTs. Implementation by local managers or government officials ensures the sustainable integration of the training into local mechanisms (policies, processes, etc.) [25]. In other words, the sustainability of a ToT initiative depends on the successful transfer of ownership to a strong local leadership (Table 5).

**Table 5:** Implementation

STRUCTURAL LEVEL	SUGGESTED CRITERIA (examples)
Individual	Evidence of training skills gained Evidence of conducting training Evidence of developing training
Organisational	Strategy to cascade (e.g. making training mandatory) Ownership (training transferred from UK partner to overseas partner)
Supra-organisational	Strategy to cascade (e.g. new educational programme) Ownership (e.g. policy) Expansion of training (e.g. into other regions/hospitals)

#### Case 4: Engaging decision makers for mentorship training in Malawi

A partnership between a teaching college of health sciences (CHS) in Malawi, a non-governmental healthcare provider and trainer of healthcare practitioners in Malawi (NGHP), and a Nursing School at a UK University (NSUK) started from an impromptu visit of a lecturer from NSUK to CHS in 2005. Both nursing schools identified reciprocal benefits from adopting the partnership approach, based on the WHO’s ‘twinning’ model between HIC and LMIC institutions [26]. Students from the UK visited Malawi for their overseas elective and, senior teaching staff from CHS visited NSUK as part of a staff development opportunity. It was soon identified by CHS staff that mentorship in practice was an important development opportunity that would enhance student clinical placement experience and retention. Mentorship of student nurses during clinical placements was not a formal element of nursing education in Malawi but conducted on an ad-hoc basis. Students arriving in their placements lacked appropriate supervision and were not able to integrate well in the workplace and gain valuable learning opportunities, and this contributed to increasing levels of attrition. The partnership set out, therefore, to establish a mentorship training programme for clinical teachers and tutors at CHS and the other nursing schools run by the NGHP. The role of these tutors was to train nurses at a number of Malawian colleges and hospitals in the principles of mentoring. The mentoring training programme was rolled out to the seven hospitals in Northern Malawi, and by the end of the project a number of clinical teachers and tutors had completed ToT courses and cascaded the training to approximately 355 nurses and clinical tutors who went to become student trainers and mentors.

Critical to the sustainability of the ToT was the partnership’s effective advocacy with senior decision makers. The partnership decided to invite the Registrar of the Malawi Nursing & Midwifery Council (MNMC) and the Chief Nursing Officer of Malawi for a visit to the UK, where they saw for themselves how mentoring in practice was embedded in the nursing curriculum. The visit was pivotal to promote mentoring as an effective method of facilitating learning in practice, and the MNMC, responsible for setting the standards for nursing education in the country, recognised the value of mentoring and became enthusiastic advocates for its inclusion in the nursing curriculum. The MNMC hosted a National Nursing Conference on Mentorship that was open to all schools of nursing in the country and attended by the Deputy Prime Minister and key members of the Malawian Ministry of Health. Following on from this conference, the MNMC developed a set of national competencies and standards for nursing and midwifery education which includes a requirement for all student nurses on clinical placements to be supervised by a mentor. Such requirement created a need for the mentorship training programme to be adopted across the whole of Malawi, thus ensuring the long-term continuity of the training.

The partnership strategically influenced MNMC and other key policy makers to appreciate the importance of the training programme. Through early engagement, the programme became owned and shaped by local actors. The UK partner stated: *“One of the most important lessons learnt is the importance of being aware of the political landscape and being politically astute to take advantage of emerging opportunities and combined with that is the ability to be flexible with the project plan.”*

### Nurture and development

Whilst it is important for trainers/educators to have the right set of technical competencies, ToT courses must also help individuals develop soft skills, i.e. attitudes and behaviours that enable learning: ability for constructive criticism, empathy, flexibility, etc. In this regard, having the opportunity to conduct training under the supervision of a MT is key to the success of a ToT [9]. ToT are usually short workshops embedded into larger training programmes [e.g. [27]]. This limits the opportunities for LTs to conduct supervised training. It also means that emphasis is generally on the initial transfer of skills from MTs to LTs. However, the sustainability of the ToT depends not only on LTs being trained (which includes opportunity for MT supervision and feedback) but crucially on preventing de-skilling over time. Sustainable upskilling of the workforce requires opportunities for CPD to be integrated into the long-term vision of the partnership (Table 6). This should include not only the ongoing one-to-one peer-support that is at the heart of GHP but should extend to providing access to relevant literature, further courses, and networking opportunities beyond the partnership. Nurturing LTs should not be limited to the initial training but should be gradually integrated into local mechanisms with decreasing support from the partnership.

**Table 6:** Nurture and Development

STRUCTURAL LEVEL	SUGGESTED CRITERIA (examples)
Individual	Are “soft” skills taught? Time to train under supervision (assessment) Engagement with CPD
Organisational	Provision of opportunities for CPD Mentoring of trainers Generation of new trainers (local ToT)
Supra-organisational	Development of formal and informal networks (North-South or South-South) Provision of learning resources (e.g. access to books, journals, etc.)

#### Case 5: Nurturing educators in Ghana through reflective practice

The Kintampo Project (KP) is a GHP established in 2007 between a college of health in Kintampo (CoHK), Ghana, and an NHS Foundation Trust in the UK (NHSFT) to create an entirely new workforce of mental health practitioners to work in rural and at the time underserved parts of Ghana (www.thekintampoproject.org*)*. This was seen as necessary to compensate for the small number of mental health professionals - approximately 10–20 psychiatrists and 100 community registered mental health nurses (RMNs) to serve a population of circa 24 million people. The new cadre would be entirely community-based and focus particularly on rural areas, thus helping shift the focus of care away from large psychiatric hospitals and into the community where it was most needed.

The challenge for the project was to establish a predominantly practice-oriented and reflective educational programme for practitioners that did not exist in Ghana at the time. Seventy percent of the teaching time would be spent in practice and the remainder at the College where students would prepare for and reflect on their practical experiences. Given the very practical and reflective nature of the programme, adequate mentorship was key. This therefore called for a strong emphasis on teacher development, on the principle that “*there can be no educational development without teacher development*” [28]. The first mentors were recruited among practicing psychiatrists and community based RMNs who attended ToT workshops. These mentors were therefore expected to train a new and different kind of workforce whose remit was still to be delineated. For this reason, developing their skills and identity as educators was considered paramount to the success of the programme.

Once the project was agreed and sanctioned at national level, the UK partner provided expertise to develop the educational programme and to enhance the educators’ educational practice using evidence-based teaching approaches that were firmly grounded in educational thinking. To this end, groups of CoHK senior educators spent short periods at a UK university to support their development needs. The UK team introduced the concept of reflective learning as a deliberate and active process by which learners pursue “active, persistent and careful consideration of any belief or supposed form of knowledge in the light of the grounds that support it, and further conclusions to which it leads” [29].

Over time, the newly trained community of mental health workers have became part of the teaching staff and many of the original ‘trainer’ RMNs have now been replaced by the most able teachers amongst the new workforce. Some have been appointed as teaching assistants at CoHK. In addition, educational (ToT) workshops were held in Kintampo annually between 2008 and 2014 supported by UK grants. NHSFT’s role was always to facilitate, not to deliver, and their input gradually reduced. By 2015–16 CoHK staff and the graduated mental health workers themselves were independently running the programme. The ToT workshops latterly became a combination of ToT and CPD for the new workforce and this kept more practitioners involved, avoided exclusivity and galvanized the interest of those who would like to focus more strongly on the trainer aspect of their service work**.**

## Conclusions

ToT is widely used as an efficient and effective approach to address the shortage of healthcare workers in LMICs through upskilling and to improve their performance, commitment and ultimately retention. Yet, we know little about the conditions necessary for ToT to achieve these positive results. Our findings highlight that whilst a variety of approaches to ToT exist, there are a number of limitations that can prevent this model of capacity-building from being both effective and sustainable. Whilst most partnerships strive to ensure the long-term sustainability of the training by focusing on adequate training provision and support of local trainers, the wider issues that can facilitate or prevent the continuation of training are not always considered. These can include the negotiation of workloads and financial responsibilities, the effective engagement with decision makers through alignment of objectives and post-project support.

Our findings, together with the THET’s vast experience of supporting partnerships and the analysis of the literature informs the conceptual framework we propose here. The TRAIN framework offers a blueprint against which ToT interventions can be mapped, allows a structure of requirements at various levels that allows individual capabilities and opportunities for training to translate into the achievement of a long-term training cascade. Although the framework will be valuable to health partnerships as a guidance in the design and operationalisation of ToT, we recognise that in practice it may be difficult to implement it in its entirety. Of the five constitutive elements, it may be more feasible to address Talent, Implementation and Nurture and Development as these are more within the reach of a small-scale partnership. Resources and Alignment may be much more demanding because they require ToT to be embedded at individual, organisational and supra-organisational level and projects may not have the scope to influence decision making at all these levels.

The TRAIN framework is intended as a starting point for further refinements and discussions about the factors affecting capacity building strategies based on training cascades. The key message underlying the framework is that the sustainability of a cascade depends on a number of “constitutive elements” being present or developing at different structural levels during the course of the project. These elements bring into focus the various conversion factors that are crucial to transform “capabilities”, i.e. the opportunities that ToT opens to LTs, into functionings, i.e. the actual achievements of a training cascade that ultimately result in the upskilling of the workforce and the improvement of health outcomes in LMICs.

## Additional file

Additional file 1: Global Health Partnerships. Table containing details of the dataset used in the analysis. It comprises 15 medium-size Global Health Partnerships supported by THET’s Health Partnership Scheme with funding from the UK government’s Department for International Development (DFID). The focus was on medium-size projects as they were deemed paradigmatic of GHP and because of the unfeasibility of a large-scale analysis of all THET-funded GHP. Only projects with ToT activities were included in the analysis. (DOCX 23 kb)
