# Supplementary material for: A conceptual framework for training of trainers (ToT) interventions in global health
Source: Global Health. 2018 Oct 22;14:100. doi: 10.1186/s12992-018-0420-3 (PMC6198384; doi:10.1186/s12992-018-0420-3)
Supplement: Supplementary file 1 — Global Health Partnerships. Table containing details of the dataset used in the analysis. It comprises 15 medium-size Global Health Partnerships supported by THET’s Health Partnership Scheme with funding from the UK government’s Department for International Development (DFID). The focus was on medium-size projects as they were deemed paradigmatic of GHP and because of the unfeasibility of a large-scale analysis of all THET-funded GHP. Only projects with ToT activities were included in the analysis. (DOCX 23 kb) [file 12992_2018_420_MOESM1_ESM.docx]

Additional file 1.

List of medium-size Global Health Partnerships funded by THET's Health Partnership Scheme. The focus was on medium-size projects as they were deemed paradigmatic of GHP and because of the unfeasibility of a large-scale analysis of all THET-funded GHP. Only projects with ToT activities were included in the analysis, resulting in a dataset of 15 projects.

| **Project** | **ToT** | **Status** | **Country** | **Health Theme** | **HIC Partner** | **LMIC Partner** |
| --- | --- | --- | --- | --- | --- | --- |
| Developing specialist eye care services for the people of northern Zambia | no | Complete | Zambia | Eye health | NHS Foundation Trust | Central Hospital |
| Developing a centre of excellence in Tanzania for the therapeutic management of violence and aggression | **yes** | Complete | Tanzania | Mental Health | NHS Foundation Trust | Hospital and Nursing School |
| Training and capacity development for colposcopy and cervical pathology reporting in Kathmandu, Nepal | **yes** | Complete | Nepal | Sexual & Reproductive Health | University Hospitals NHS Trust | Maternity Hospital |
| Training peer support workers (PSW's) to support community mental health in urban Uganda | no | Complete | Uganda | Mental Health | NHS Foundation Trust | Hospital |
| Training of a multi-disciplinary team to support the launch of a burns unit in Tanzania | no | Complete | Tanzania | Accident & Emergency Health | Healthcare NHS Foundation Trust | Medical Centre |
| Health link "care" project | **yes** | Complete | South Sudan | Maternal & Newborn Health | Healthcare NHS Trust | Hospital (& other Leads) |
| Reducing health harm caused by alcohol | **yes** | Complete | Ghana | Mental Health | NHS Highland | Regional Health Service |
| Reducing newborn mortality with staff training, guidelines and respiratory and nutritional support in Rwandan hospitals | no | Complete | Rwanda | Maternal & Newborn Health | University | University Teaching Hospital |
| Fistula Training Link | **yes** | Complete | Uganda | Sexual & Reproductive Health | Hospital | Health Care Complex |
| Utilising a 'training the trainers' approach to developing teaching skills of Malawian educators and nurses | **yes** | Complete | Malawi | General health | University | College of Health Sciences |
| Link primary trauma course | **yes** | Complete | Uganda | Accident & Emergency Health | University Hospital NHS Foundation Trust | Faculty of Medicine & Regional Referral Hospital |
| Stroke partnership project to develop multi-disciplinary management of patients with stroke | **yes** | Complete | Ghana | Non-communicable Disease | NHS Foundation Trust | Teaching Hospital |
| Health system strengthening via WHO-AIMS in Ghana and building expertise in quality mental health informatics | no | Complete | Ghana | Mental Health | NHS Foundation Trust | College of Health |
|  | no | Failed | Bangladesh | n/a |  |  |
| Localising tools and training to improve maternal and perinatal outcomes in Bulawayo and beyond | **yes** | Complete | Zimbabwe | Maternal & Newborn Health | NHS Trust | Hospital |
| Building professional capacity to improve child health in Palestine | **yes** | Complete | Occupied Palestinian Territories | Child Health | Professional Association | Medical School |
| Improving maternal and child health care in remote rural Nepal by supporting primary care workers | no | Complete | Nepal | Maternal & Newborn Health | NHS Foundation Trust Hospital | District Health Office |
| Obstetric anaesthesia courses for the whole of the Ugandan anaesthetic workforce | **yes** | Complete | Uganda | Maternal & Newborn Health | Professional Association | Professional Association |
| Introduction of “Training the Trainer” (TTT) Courses to enhance surgical training in West Africa | **yes** | Complete | Nigeria, Ghana, Senegal | General health | Professional Association | Professional Association |
| Bangladesh Child Cancer Project | no | Ongoing | Bangladesh | Child Health | University College Hospital | Medical University |
| Post Traumatic Limb Reconstruction Fellowship for Palestinian Surgeons | no | Complete | Palestine | Accident & Emergency Health | University College Hospital | Hospital |
|  |  |  |  |  |  |  |
| Establishing continuous CPD for recently qualified Community Mental Health care workers in Ghana | **yes** | Complete | Ghana | Mental Health | NHS Foundation Trust | College of Health |
| Reducing the burden of respiratory and other chronic diseases in rural Ethiopia | **yes** | Ongoing | Ethiopia | Non-communicable Disease | University Hospital NHS Foundation Trust | University Hospitals |
| Endoscopic therapy and TTT to sustainably prevent deaths from Acute Upper GI Bleed in Malawi | no | Complete | Malawi | General health | School of Endoscopy | Clinical Investigation Unit, Central Hospital |
| The implementation of the first Paediatric Nursing course in Zambia | no | Complete | Zambia | Child Health | University | University Teaching Hospital |
| Reducing neonatal mortality and maternal and paediatric infection through improved patient safety in Rwanda | no | Complete | Rwanda | Maternal & Newborn Health | University | University Teaching Hospital |
| Maternity Train the Trainers | **yes** | Complete | Kenya | Maternity and newborn care | Teaching Health Board | Government District Hospital |
